# A tripartite evolutionary game analysis of sports data rights protection from the perspective of stakeholder

**DOI:** 10.1371/journal.pone.0292914

**Published:** 2023-11-16

**Authors:** Xiaoyu Li, Xinyan Guo

**Affiliations:** School of Economics and Management, Chengdu Sport University, Chengdu, Sichuan, China; Al Mansour University College-Baghdad-Iraq, IRAQ

## Abstract

The amount of data in the globe is increasing at an exponential rate, and the role of digital technology and data elements in the modern growth of China’s sports business is becoming more significant. Disputes over sports data rights have grown in the international arena, with a special focus on how to preserve the sports data rights of key stakeholders. This study begins from the standpoint of stakeholders, constructs a tripartite evolutionary game model of the government, sports enterprises, and the public around the issue of sports data rights protection, investigates the behavioral evolutionary characteristics of each stakeholder and its stabilization strategies, simulates and analyzes the evolutionary stabilization strategies of the stakeholders in various situations using MATLAB, and proposes a compatible institution. According to the findings of this study, the cost of sports data governance, the cost of sports data sharing, the degree of sports data compliance use, the government incentive system, and the government regulation mechanism are the important elements impacting each stakeholder’s behavioral decision-making. Based on the foregoing, the study proposes corresponding institutional strategies for governments to respond to the issue of sports data rights, which will be more conducive to the safe use of data for development and humanitarian action, as well as contribute to global sustainable development.

## Introduction

With the rapid development of digital technology, data has emerged as a new and crucial production factor. However, because there are different types of data and different data characteristics, data protection issues still face many practical dilemmas, such as sports data being more difficult to anonymize or de-identify than general data, and because a large portion of sports data originates from athletes’ data, de-identifying the athletes’ sports personal data may weaken the original value of some sports data. As a result, the issue of privacy of sports data and the issue of ownership of sports data begin to be highlighted.

Concerning the protection of sports data rights, Chinese and foreign scholars have mostly focused on the discussion of the protection of the right to webcasting of sports events [1]. Chinese scholars believe that the dilemma in justice is that it is impossible to characterize the behavior of network broadcasting of sports events, and it is difficult to regulate the behavior of network broadcasting through the existing institutional framework [2, 3]. Foreign scholars have proposed a data technology-based legal protection model for the network broadcasting rights of sports events, which realizes the modeling of the legal protection of broadcasting rights by establishing a legal protection risk evaluation index system [4]. With the explosive growth of data volume around the world, the rational utilization and development of sports event data has attracted the attention of scholars from all walks of life. The effective legalization of sports betting has multiplied the value of sports data, which has become an integral part of sports betting operations, and the question of how to acquire, manage, monitor, and purchase this sports data has arisen. Professional league commissioners have argued that they should have property rights in sports events, and are concerned that legislation governing sports data ownership does not limit unauthorized collection and use. In response, scholars have proposed federal legislation to address sports data rights and effectively balance the interests of leagues and sports betting [5]. In addition to the rights protection dilemma faced by sports event data, sports personal data is also at risk of privacy leakage. Smart wearable devices often collect and share user-generated trajectory data to social apps to provide health services and recommendations, which creates the problem of privacy leakage of users’ personal data, while optimization algorithms may be able to protect users’ data [6]. It can be seen that the complexity of the problems faced by the protection of sports data rights.

Sports data often involves a variety of types of data such as sports event data, sports personal data, sports public data, etc., the generation and application of which involves several subjects, such as government sports departments, sports enterprises, sports social organizations, athletes, and consumers. The different interests of these relevant subjects make conflicts of interest often arise and further aggravate the reality of the dilemma of sports data rights protection [7]. Sports data often involves various types of data, such as sports event data, sports personal data, sports public data, etc., and its generation and application involve multiple subjects, such as governmental sports departments, sports enterprises, sports social organizations, athletes, consumers, etc., and the interests of these related subjects are different, which makes conflicts of interest often appear and further aggravates the reality of the dilemma of sports data rights protection [1]. The solution to this dilemma focuses on sorting out the various data subjects and their intertwined relationships in sports data rights protection and identifying the key influencing factors. The stakeholder strategic management framework proposed by Freeman can effectively solve the problem of identifying sports data subjects. The construction of the evolutionary game model can effectively respond to two questions: 1) What are the key influencing factors of the data rights protection dilemma, and how do these influencing factors affect the decision-making of the relevant stakeholders? 2) How to formulate corresponding institutional strategies to change the sports data rights protection dilemma, to effectively incentivize the willingness of the enterprises and the public to share?

Existing studies have mainly used the stakeholder analysis framework to study data management [8], users’ personal data rights [9], and open government data [10]. There is a gap between Chinese and foreign research on the use of the stakeholder analysis framework for sports data rights protection, and the applicability of the stakeholder analysis framework will be discussed in the following section. In the process of sports data utilization and sharing, there are usually problems of information asymmetry, technological differences, and cognitive limitations, so it is difficult for sports data subjects to satisfy complete rationality as participants [11]. At the same time, the circulation and utilization of sports data is a long-term dynamic process, a dynamic gaming process in which participants continuously adjust their strategies based on past practices and experiences. According to the existing research findings, many scholars have applied the evolutionary game analysis method to the discussion of data sharing, data personal information protection, government data quality management, and other issues [12, 13]. While evolutionary game analysis is mainly used in the field of sports in the collaborative governance of public sports services, school sports governance, and other aspects [14, 15], there is a lack of application and analysis in the protection of sports data rights and interests. Therefore, the study applies the stakeholder strategic management framework to identify the stakeholders of sports data rights protection and comprehensively evaluate their identities and rights. Second, the stakeholder relationship is interpreted and analyzed, i.e., the actual and potential behaviors of the stakeholders of sports data rights protection are clarified. Third, the evolutionary game model of sports data rights protection is constructed based on the analysis of stakeholder relationships, and the evolutionary simulation analysis is carried out by combining the real situation and existing research data to further verify the correctness and validity of the results of the evolutionary game model. Finally, based on the simulation results of the evolutionary game model, the institutional strategy for the protection of sports data rights is proposed.

This study systematically analyzes the issue of sports data protection from the perspective of stakeholders, and introduces the dynamic evolution game into the stakeholder analysis framework, aiming to more objectively and present the realistic dilemmas of sports data protection, and the institutional strategy of sports data protection based on this. This will enrich the existing research paradigm in the field theoretically, provide a reference for governmental governance in practice, optimize the circulation and utilization of sports data, and contribute to the sustainable development of the world.

## Related work

### Structural analysis of stakeholders

According to the strategic stakeholder management framework proposed by Freeman, identifying stakeholders is the primary issue, i.e., identifying the stakeholders of sports data rights protection and evaluating their identity and rights comprehensively [16]. It is particularly important to identify and analyze the stakeholders involved in sports data since sports data not only includes data directly related to sports but also data indirectly related to sports. From the development of the sports industry in China during the 13th Five-Year Plan period, the proportion of the sports service industry has been increasing, and gradually becoming the leading industry in the sports industry, while the proportion of sports manufacturing industry in the sports industry has been decreasing. Among them, the sports service industry includes core industries such as sales, rental, and trade agency of sports goods and related products; sports fitness and leisure activities; sports competition and performance activities; and sports education and training; while the sports manufacturing industry includes manufacturing of sports goods and related products. The sports data discussed in this study mainly focus on sports data generated by the sports service industry, such as mass sports data generated by the sports fitness and leisure industry, athletes’ data, and event organizers’ data generated by the sports competition and performance industry, and corporate sports data or consumer sports data generated by the sales, rental, and trade agency of sports goods and related products, etc. Therefore, the stakeholder analysis of sports data rights protection will be based on this analysis. Therefore, the stakeholder analysis of sports data protection will also be conducted based on this.

#### Government

For the protection of sports data rights in the sports service industry, the government plays multiple roles as a leading player, regulator, and supervisor. The government improves the scope and content of sports data rights protection through policy-oriented, effectively revitalizes the scale of sports data, and enhances the value of sports data, while preventing and controlling the negative externalities of sports data usage. However, it is unrealistic to rely solely on the government to solve the problem of sports data rights protection. On the one hand, if the government, as a supplier of public goods, does not manage effectively, it will easily lead to the phenomenon of "tragedy of the commons" or "anti-tragedy of the commons". On the other hand, regional economic, social, and cultural differences, different policy priorities, time lags in policy implementation, and differences in the goals of local governments make the phenomenon of "government failure" appear frequently.

#### Sports enterprises

In the protection of sports data rights and interests, sports enterprises are both data processing subjects and data source subjects, and sports enterprises often have a large amount of internal data. Once these data are leaked, enterprises may suffer huge asset losses. Therefore, sports enterprises, as important producers and users of sports data, are the main stakeholders of sports data rights protection and bear important responsibilities for the protection of sports data rights and interests. However, from the perspective of economics, the behavioral goal of enterprises is to pursue profit maximization, and taking too much responsibility for sports data rights protection will increase their operation cost to a certain extent and even affect their revenue, which leads to sports enterprises being reluctant to take the corresponding responsibility when facing the issue of sports data rights protection, and even appearing "speculation" which is detrimental to fair competition. This has led to the reluctance of sports enterprises to take responsibility for the protection of sports data, and even to "speculation" that undermines fair competition.

#### Public

The public is the consumer and supervisor of various sports products and services, as well as the beneficiary and victim of sports data dividends, and is one of the important subject elements of sports data rights protection. Personal privacy and public safety issues arising from data security often ultimately point to data subjects, including the public. For example, the National Security and Personal Data Protection Act of 2019, the California Consumer Protection Act of 2020, the EU General Data Protection Regulation, the Personal Data Act of 2015, and other relevant legislation emphasize the protection of personal data and privacy. Among the multi-stakeholder subjects of sports data rights protection, the public plays a certain guiding role. Firstly, the public is the focus of sports data rights protection, and the interest preference of the public will directly affect the goal and direction of sports data rights protection, if the interests of the public are not properly dealt with in the policy formulation, deviations will inevitably occur in the future policy implementation, thus generating certain negative social externalities. Secondly, the social public covers a wide range, and the boundary is blurred, and even intersects with other stakeholders, such as the government, enterprises, and social organizations, whose members also belong to the social public and are affected by the effect of sports data rights protection, thus, the preferences and interests of the social public are satisfied to a certain extent, which also affects the preferences of other stakeholders.

#### Social organizations

In China, social organizations are an important part of the multi-governance structure of sport, mainly as an important receiver of the transfer of government functions. Social organizations mainly play the role of organizers in sports data rights protection, and their main responsibilities are to promote the effective allocation of sports resources and to coordinate the relationship among various stakeholders through non-government coercive means. However, the non-profit nature of sports social organizations in China often restricts them from performing public service functions such as allocating sports resources, protecting the interests of the public, and assisting the government in governance. In addition, it is difficult to implement tax incentives, which makes sports social organizations still depend on the government’s financial support, so sports social organizations are still in the state of "half-government and half-people" [17].

### Relationship analysis of stakeholders

The rapid development of digital information technology has triggered a digital transformation of social production methods, and data has become a "necessity" for economic and social production and life. For example, the Fast Stats health data platform allows users to view various types of electronic health data online in real-time via cell phones and share data through other social media channels such as Facebook and Twitter [18]; ClubView’s financial data platform provides real-time business performance analysis and expert insights into revenue mix, investment priorities and expansion opportunities for soccer clubs, and currently supports 114 clubs in the UK, including all English Football League teams and the entire Scottish Premier League [19]. Sports data can be organically integrated with all factors of production and lead to productivity gains. With the continuous expansion and refinement of computing power and algorithm technology, these sports data can be processed, stored, analyzed, and mined to generate huge commercial value. These make the conflict of interest between various stakeholders increasingly prominent, forming a more complex interaction.

#### Government and sports enterprises

The relationship between the government and sports-based enterprises is mainly one of cooperation and regulation. On the one hand, the cooperation between the government and sports enterprises can further explore the value of sports public data. Generally speaking, the government holds a large amount of sports public data, including data collected and generated in the process of performing its duties or providing public services according to the law; data on public stadiums and sports facilities; data on public welfare sports events or sports activities; and data on the sports industry. These sports public data are considered to have the duality of "governance factors" and "production factors", but their economic and social values as "production factors" are often neglected. Sports public data are considered to be able to enter the market as a factor of production, provided that national security is not involved [20]. However, the level of government financial resources and the nature of projects in China are complex, and although government-enterprise cooperation offers the possibility to promote the realization of the value of sports public data, it is necessary to choose the most appropriate government-enterprise cooperation model according to the specific situation [21].

On the other hand, the compliant use of data by sports enterprises needs to be regulated by the government. Sports enterprises usually have two types of data, one is the data collected or generated in the independent operation of sports enterprises, such as sports event data obtained by sports event organizers through running games; athletes’ data obtained by sports clubs in daily training or competitive games. Another type of data is data generated after processing third-party data, such as sports data products generated by professional data service platforms after processing and analyzing event data. However, in the absence of relevant laws and regulations, the non-compliant use of data by sports enterprises has frequently emerged, such as the illegal collection and use of personal information unrelated to the services they provide by 34 sports and fitness apps, including Keep, Codoonsport, Zepp Life, and JOYRUN. With the increased reliance of sports enterprises on data, the compliant collection and utilization of data has become critical, and sports enterprises often need certain financial, human, and material investments to carry out internal governance and build data compliance systems. However, facing high operating costs, sports enterprises often tend to reduce costs to obtain higher profits and thus achieve their business goals. Therefore, sports-type enterprises tend to adopt a passive governance attitude in data compliance construction, and the only way for enterprises to passively participate in data governance is for local governments to strengthen governance subsidies or regulations.

#### Government and the public

The relationship between the government and the public can be described as bidirectional management. In public administration, the public often plays two roles, one is the object of public management and the other is the participant of public management. According to public management theory, the objects of public management include citizens, social organizations, and enterprises, and these governed people need to fulfill their civic obligations and accept the government’s management. In the protection of sports data rights, the government’s governance model of sports data often adopts the government-led-cooperation model, and the government still retains macro control over the security of sports data, including the formulation of relevant laws and regulations and policy documents to regulate the collection and utilization of sports data. And the government also attaches importance to extensive cooperation with other organizations (enterprises and the public), to achieve diversified and integrated governance goals for sports data. However, in modern public management activities, public management targets as stakeholders have changed from being managers to participants, i.e., they are no longer passive recipients but participate in the process of making, implementing, and monitoring public policies and public management. At present, there are more than 800,000 network sports organizations active in urban and rural grassroots communities in China, and these social organizations are constantly reshaping the public organization network of national fitness in China, and actively exploring the construction of a public service organization network of national fitness and intelligent service platforms have become the focus of the government’s work [22].

#### Sports enterprises and the public

In the protection of sports data rights, the relationship between sports enterprises and the public can be regarded as service and sharing, where the public is an external social group with direct influence and role in enterprises. First of all, sports products or services are the carriers of all the activities of sports enterprises, and what customers ultimately consume are sports products or services, so the relationship between sports enterprises and the public can be regarded as a service and being served. For example, fitness and leisure, competition and performance, stadium services, and sales of sports-related goods are directly directed to sports consumers; the training courses, exercise guides, and health monitoring provided by sports software ultimately serve the sports consumers who use the software; the sports event data analysis company, Trajektory, provides data analysis services for sports organizations, etc. [19]. Secondly, the willingness and extent of data sharing by the public to sports enterprises will directly affect the circulation and utilization of relevant data, which in turn affects the development and benefits of sports enterprises. In the digital era, sports data has become a key element to drive the business growth of sports enterprises, and sports enterprises can not only drive core business development through data analysis and data visualization analysis but also indirectly drive related business development, where do these sports data come from? Usually, sports business data of sports enterprises include self-generated data, independently collected data, and data supplied by third parties, and the acquisition of the latter two types of data often depends on the willingness of relevant data subjects to share.

## Model formulation

### Model assumptions

To achieve the full circulation and reasonable use of sports data, it is necessary to realize the game through the dynamic game of multiple stakeholder groups, specifically the game between the government, sports enterprises, and the public, therefore, the following assumptions are made. For the readers’ convenience, the main symbols used in the paper are listed in Table 1.

**Table 1:** Main symbols used in the paper.

Symbol	Description
**R1**	The benefits received by the public in the primary market
**R2**	The benefits received by the public in the secondary market
**R3**	The benefits received by the sports enterprises in the primary market
**R4**	The benefits received by the sports enterprises in the secondary market
**C**	The cost of strict management
**C1**	The costs incurred by the public in authorizing sports enterprises to make secondary use of sports data
**C2**	The cost of time, money, and manpower, including the input cost of acquiring and creating sports data, incurred by sports enterprises in applying big data technology or services to process and analyze sports data from the public
**C3**	The social public paying litigation costs
**J**	The subsidy used to incentivize sports enterprises to actively participate in the governance of data
**nC**	The cost of government management
**nJ**	The subsidy funds are recovered
**L**	The losses caused to the public by the illegal use of data by sports enterprises
**F**	The fine from government regulators
**I**	The compensation from sports enterprises
**r1**	The individual gain coefficient influenced by the information sensitivity
**l1**	The risk of loss of personal privacy compromised by the information sensitivity
**r2**	The individual gain coefficient influenced by the level of data utilization
**l2**	The risk of loss of personal privacy compromised by the level of data utilization
**n**	The probability of government random checks on sports enterprises
**s**	The information sensitivity of the public
**v**	The level of sports data utilization by sports enterprises to the social public
**m**	The reputation level of sports enterprises in data management

#### Assumption 1

There are three participants in the game of data rights protection: government (G), sports enterprises (S), and the public (P), all of whom have limited rationality. The government incentivizes enterprises to collect, utilize, and secondary process sports data, and supervises and manages this process. The sports data held by sports enterprises usually come from the public, such as consumers’ sports data and athletes’ sports data, etc. The processing, utilization, and management of these data will be directly related to the rights and interests of the public. The public’s willingness to share their own sports data will also directly affect the circulation and utilization of sports enterprises’ data.

#### Assumption 2

The compliant use of sports data by sports enterprises requires government regulation. At this point, the government’s strategy space is strictly managed z and loosely managed 1-z. The cost of strict management is C and the subsidy J is used to incentivize sports enterprises to actively participate in the governance of data. In the case of lax government management, sports enterprises may govern negatively, while the government conducts random checks on sports enterprises with probability n. The cost of government management is nC, and the subsidy funds are recovered nJ. Sports enterprises decide to hold the strategy of positive governance as x and the strategy of negative governance as 1-x after measuring their governance benefits and governance costs. The social public strategy is chosen as willing to share or unwilling to share, denoted as y, respectively. 1-y.

#### Assumption 3

Generally, the market in which the conditions for the collection and use of personal data are agreed upon is called the primary market [8]. For example, sports-based enterprises collect sports data from the public through server control and search engine traces, and process, model, and calculate these data to provide the public with the corresponding services, a process that occurs under the premise that the public agrees to the relevant privacy agreements and other form contracts. If the public does not accept the rules set by the sports enterprises from the beginning, it means that the sports enterprises do not obtain the right to use the corresponding sports data and the public cannot obtain the corresponding services provided by the sports enterprises. Thus, let the benefits received by the public and sports enterprises in the primary market be R1 and R3, and the returns in the primary market are fixed and not disturbed by other factors.

#### Assumption 4

The secondary market has developed under the secondary use of personal data. With the rapid development of digital technology, sports enterprises have started to move from analyzing sports data with a predetermined purpose, manner, and scope to processing sports data outside the predetermined scope, i.e., discovering new laws and uses from the unknown mass of data. Therefore, sports enterprises are not able to inform the public about the use of sports data in advance (i.e., it is not specified in the initial user privacy agreement), and to mine and reuse the data in the secondary market, sports enterprises need to obtain the authorized consent of the public. Therefore, let the benefits received by the public and sports enterprises in the secondary market be R2 and R4, respectively, and the costs incurred by the public in authorizing sports enterprises to make secondary use of sports data (e.g., the time cost of reading lengthy privacy protection agreements), denoted as C1, and the cost of time, money, and manpower, including the input cost of acquiring and creating sports data, incurred by sports enterprises in applying big data technology or services to process and analyze sports data from the public, is recorded as C2.

#### Assumption 5

The Guidelines for the Protection of Personal Information in Information Security Technology Public and Commercial Service Information Systems classify personal information into general information and sensitive information, general information is information that the individual has tacitly agreed to be collected and used. Whereas sensitive information is the information that requires the explicit authorization and consent of the individual. Therefore, let the information sensitivity of the public be s. Sensitivity is positively correlated with the benefit and negatively correlated with the risk of damage. The higher the sensitivity the greater the gain coefficient r1 to the individual, and the greater the risk of loss of personal privacy disclosure l1. For example, the personal biological data of NBA players often belong to their sensitive data, and NBA leagues often rely on third-party data enterprises to process and analyze player data, which provides great support for league operations, management, and decision making, but there is also the risk of loss of sensitive data being leaked.

#### Assumption 6

Let the level of sports data utilization by sports enterprises to the social public be v. The high mobility and strong generality of sports data determines the high level of data utilization, which brings considerable data revenue to sports enterprises and also makes the benefit coefficient r2 of the social public increase, at this time, the greater the risk of damage l2 of the social public’s sports data being leaked. The reputation level m of sports enterprises in data management will influence the willingness of the social public to provide sports data. A good reputation level such as establishing data security management mechanisms and encrypting and desensitizing sports data will motivate the social public to actively share data.

#### Assumption 7

When the social public refuses to share sports data, the sports enterprises’ illegal use of the data will cause certain losses to the social public L, such as phishing attacks or financial frauds caused by the leakage of NBA fans’ data, and price discrimination problems caused by the leakage of sports consumers’ data. When the social public refuses to share sports data, the probability of sports enterprises using the data illegally is n. Then, the sports enterprises that are found will face penalties from both the government and the social public: one is the fine F from government regulators, and the other is the social public paying litigation costs C3 to defend their data rights and interests through justice, to get compensation I from sports enterprises.

### Model construction

According to the model assumptions, a tripartite game payment matrix is constructed. The game payment matrix is shown in Table 2.

**Table 2:** Tripartite game payment matrix.

Gaming party				Government	
				Strict Regulation (z)	Loose Regulation(1-z)
**Sports Enterprises**	**Active governance (x)**	**Public**	**Willing to share (y)**	R3+sR4+vR4+mR4-C2+J, R1+(r1-l1)R2+(r2-l2)R2-C1, -C-J	R3+sR4+vR4+mR4-C2+J, R1+(r1-l1)R2+(r2-l2)R2-C1, -nC-J
			**Refuse to share (1-y)**	R3+sR4+vR4+mR4-n(F+I)-C2+J, R1-L+n(I-C3), -C-J	R3+sR4+vR4+mR4-n(F+I)-C2+J, R1-L+n(I-C3), -nC-J
	**Negative Governance (1-x)**	**Public**	**Willing to share (y)**	R3, R1-C1, -C	R3, R1-C1, -nC
			**Refuse to share (1-y)**	R3, R1, -C	R3, R1, -nC

In the tripartite game payment matrix, sports enterprises have data dividends from active data governance, such as government subsidies and additional profits from sharing data with the public, but also negative data governance attitudes, i.e., opportunistic behavior to reduce costs and risks. Strict government regulation can be costly in terms of effort and cost, and information asymmetry is the main dilemma that hinders government governance. Therefore, the government may adopt random-checking regulations to reduce regulatory costs, improve regulatory efficiency, and create deterrence. The public’s willingness to share will not only affect the efficiency of sports enterprises’ use of data but also affect the motivation of sports enterprises to participate in sports data governance. If the public is willing to pay a certain cost to share sports data to obtain the services or products provided by sports enterprises, then sports enterprises are also motivated to carry out sports data governance, thus forming a virtuous cycle of development. However, the core goal of each stakeholder is to maintain their interests, thus, there may be a dilemma that sports enterprises are passive in governance and the public is not willing to share sports data. At this time, the value of sports data is not fully utilized and tapped, and the corresponding benefits and costs are relatively small. The government’s unilateral regulatory game only involves the corresponding regulatory costs and fixed subsidies to enterprises. When both sports enterprises and the public show positive attitudes, then the payment function involves all cost-benefit factors. The opportunistic behavior of free-riding occurs when sports enterprises actively govern and the public refuses to share or when sports enterprises negatively govern and the public is willing to share.

1. The expected utility of choosing active governance for sports enterprises is:
E11=yz(J-C2+R3+mR4+sR4+vR4)-z(y-1)(J-C2+R3+mR4+sR4+vR4-n(F+I))-y(z-1)(J-C2+R3+mR4+sR4+vR4)+(y-1)(z-1)(J-C2+R3+mR4+sR4+vR4-n(F+I))
  The expected utility of choosing negative governance for sports enterprises is:
E12=(y-1)(z-1)R3-z(y-1)R3-y(z-1)R3+yzR3
  The replication dynamic equation for sports enterprises is:
fx=x(x-1)[C2+nF+nI-mR4-sR4-vR4-ynF-ynI-J] (1)
2. The expected utility of the public choosing to be willing to share is:
E21=(x-1)z(C1-R1)-xz(C1-R1+(l1-r1)R2+(l2-r2)R2)-(x-1)(z-1)(C1-R1)+x(z-1)(C1-R1+(l1-r1)R2+(l2-r2)R2)
  The expected utility of the public choosing to reject sharing is:
E22=x(z-1)(L-R1+n(C3-I))-(x-1)zR1+(x-1)(z-1)R1-xz(L-R1+n(C3-I))
  The replication dynamic equation for the public is:
fy=y(y-1)[C1-xL-xnC3+xnI+xl1R2+xl2R2-xr1R2-xr2R2] (2)
3. The expected utility of the government’s choice of strict regulation is:
4. E31 = x(y-1)(C+J)-(x-1)(y-1)C-xy(C+J)+y(x-1)C
  The expected utility of the government’s choice of loose regulation is:
E32=x(y-1)(J+nC)-xy(J+nC)+(x-1)ynC-(x-1)(y-1)nC
  The replication dynamic equation for the government is:
fz=z(z-1)[-C(n-1)] (3)

## Evolutionary equilibrium analysis

### Jacobi matrix

According to Friedman’s research [23], the evolutionary stable strategy (ESS) of the evolutionary game can be judged by the eigenvalues λ of the Jacobian matrix. Therefore, the Jacobian matrix of the tripartite evolutionary game is obtained by associating Eqs (1), (2), and (3), which is calculated as:

$$\text{J}=\left[\begin{array}{ccc} \begin{array}{l} (\text{x}-1)(\text{C}2+\text{nF}+\text{nI}-\text{mR}4-\text{sR}4-\text{vR}4-\\ \text{ynF}-\text{ynI}-\text{J})-\text{x}(\text{J}-\text{C}2-\text{nF}-\text{nI}+\\ \text{mR}4+\text{sR}4+\text{vR}4+\text{ynF}+\text{ynI}) \end{array} & \text{x}\left(\text{x}-1\right)\left[-\text{n}\left(\text{F}+\text{I}\right)\right] & 0\\ \begin{array}{l} \text{y}\left(\text{y}-1\right)(\text{L}+\text{nC}3-\text{nI}-\\ \text{l}1\text{R}2-\text{l}2\text{R}2+\text{r}1\text{R}2+\text{r}2\text{R}2) \end{array} & \begin{array}{l} \left(2\text{y}-1\right)(\text{C}1-\text{xL}-\text{xnC}3+\text{xnI}+\text{xl}1\text{R}2\\ +\text{xl}2\text{R}2-\text{xr}1\text{R}2-\text{xr}2\text{R}2) \end{array} & 0\\ 0 & 0 & \left(1-2\text{z}\right)\text{C}\left(\text{n}-1\right) \end{array}\right]$$ (4)

In the game model of sports data rights protection, each participant constantly adjusts its strategy according to its vested interests to pursue the improvement of its interests and finally achieves a dynamic equilibrium strategy, namely the evolutionary stability strategy (ESS). Before determining the ESS, the equilibrium point of the evolutionary game is required. Let fx = fy = fz = 0, and get the 10 stable equilibrium points of the tripartite evolutionary game. From the evolutionary game theory, it is known that the evolutionary stable points (ESS) of the tripartite evolutionary game system satisfy all the non-positive conditions of the eigenvalues of the Jacobi matrix.

### Stability analysis

Using Lyapunov’s first method: if all eigenvalues of the Jacobi matrix have negative real parts, the equilibrium point is asymptotically stable; if at least one eigenvalue of the Jacobi matrix has a positive real part, the equilibrium point is unstable; if all eigenvalues of the Jacobi matrix have negative real parts except for those with zero real parts, the equilibrium point is in a critical state and the stability cannot be determined by the sign of the eigenvalues. The stability of each equilibrium point is analyzed, as shown in Table 3.

**Table 3:** System equilibrium points and eigenvalues.

Balancing point	Jacobian matrix eigenvalues	Real part symbol	Stability	Conditions
	λ1,λ2,λ3			
D1[0,0,0]	nC-C,-C1,J-C2-nF-nI+mR4+sR4+vR4	(-,-,-)	ESS	(i)
D2[1,0,0]	nC-C,C2-J+nF+nI-mR4-sR4-vR4,L-C1+nC3-nI-l1R2-l2R2+r1R2+r2R2	(-,-,-)	ESS	(ii)(iii)
D3[0,1,0]	C1,nC-C,J-C2+mR4+sR4+vR4	(+,-,+)	Unstable	(v)
D4[0,0,1]	-C1,C-nC,J-C2-nF-nI+mR4+sR4+vR4	(-,+,-)	Unstable	(i)
D5[1,1,0]	nC-C,C2-J-mR4-sR4-vR4,C1-L-nC3+nI+l1R2+l2R2-r1R2-r2R2	(-,-,-)	ESS	(iv)(v)
D6[1,0,1]	C-nC,C2-J+nF+nI-mR4-sR4-vR4,L-C1+nC3-nI-l1R2-l2R2+r1R2+r2R2	(+,+,-)	Unstable	(i) (ii)
D7[0,1,1]	C1,C-nC,J-C2+mR4+sR4+vR4	(+,+,+)	Unstable	(v)
D8[1,1,1]	C-nC,C2-J-mR4-sR4-vR4,C1-L-nC3+nI+l1R2+l2R2-r1R2-r2R2	(+,-,+)	Unstable	(ii)(v)
D9[x_1_, y_1_, 0]	nC-C.$\sqrt{a_{1}}/a_{2}-\sqrt{a_{1}}/a_{2}$	(-,-,+)	Unstable	(ii)(v)
D10[x_2_, y_2_, 1]	C-nC.$\sqrt{a_{1}}/a_{2}-\sqrt{a_{1}}/a_{2}$	(+,-,+)	Unstable	(ii)

Note: × indicates that the sign is uncertain; x_1_, x_2_, y_1_, y_2_ are the coordinates of the corresponding equilibrium point, which is unstable or meaningless if the conditions corresponding to the equilibrium point are not satisfied. (i) C2+nF+nI > mR4+sR4+vR4+J; (ii) C1+nI > (L+nC3)+(r1-l1)R2+(r2-l2)R2; (iii) C2+nF+nI < mR4+sR4+vR4+J; (iv) C1+nI < (L+nC3)+(r1-l1)R2+(r2-l2)R2; (v) mR4+sR4+vR4+J > C2.

#### Scenario 1

When C2+nF+nI > mR4+sR4+vR4+J, i.e., condition (i) holds, there exists a stable point D1[0, 0, 0] for the replicated dynamic system. It shows that the sum of the input costs of sports enterprises for sports data processing, the fines imposed by the government on sports enterprises for illegal use of sports data, and the compensation paid by sports enterprises after successful public litigation is greater than the sum of the revenue level of sports enterprises and government subsidies, and the strategy combination evolves to be stable at (negative governance, refusal to share, and loose regulation). At this point, sports enterprises, influenced by factors such as the high cost of sports data processing and high risk of utilization, will adopt a negative attitude to deal with the issue of sports data rights protection, while the public, faced with the risk of sports data leakage, tends to adopt a no-sharing strategy to avoid the occurrence of damage. Under this circumstance, the government’s loose regulation lacks sufficient effectiveness to effectively restrain the free-riding behavior of sports enterprises. Therefore, the government can moderately enhance the regulation increase the subsidies to sports enterprises, and influence the data utilization and management of sports enterprises by regulating the degree of regulation and financial subsidies.

#### Scenario 2

When C2+nF+nI < mR4+sR4+vR4+J and C1+nI > (L+nC3)+(r1-l1)R2+(r2-l2)R2, i.e., conditions (ii) and (iii) hold, there exists a stable point D2[1, 0, 0] for the replicated dynamic system. It shows that when the sum of the level of revenue and government subsidies of sports enterprises is greater than the sum of the input cost of sports data processing by sports enterprises, the penalty paid by the government to sports enterprises for the illegal use of data, and the compensation paid by sports enterprises after successful lawsuits by the public, and the cost paid by the public to authorize sports enterprises to make use of sports data is much greater than the profit they make after sharing sports data, and the strategy combination evolves to be stable (active governance, refusal to share, and loose regulation). In this case, sports enterprises can enjoy the dividends of sports data utilization and management, such as government subsidies, data profitability, and other operational cost reductions, and thus adopt an active data governance strategy. Even so, the public still chooses to reject the sharing strategy when faced with high licensing costs, which not only affects the secondary use of data but also affects the motivation of sports enterprises. Although loose government regulation can improve regulatory efficiency, it can easily create "those who escaped punishment", making sports enterprises "avail themselves of loopholes" and violate the rights and interests of the public in sports data. Therefore, the government must strengthen regulation, while sports enterprises should reduce the cost to the public in the process of authorizing data and avoid violations.

#### Scenario 3

When C1+nI < (L+nC3)+(r1-l1)R2+(r2-l2)R2 and mR4+sR4+vR4+J > C2, i.e., conditions (iv) and (v) hold, there exists a stable point D5 [1, 1, 0] for the replicated dynamic system. It shows that the strategy combination evolves stably with (active governance, willingness to share, and loose regulation) when the profit gained by the public after sharing sports data is greater than the cost paid by them, and the benefit gained by sports enterprises is greater than their cost of processing sports data. In this case, sports enterprises can obtain enough benefits including government subsidies, profits from sports data, and other operational cost reductions, so they adopt the "active governance" strategy; the public can enjoy sports data products or services provided by sports enterprises without paying too much licensing costs, so they tend to adopt the "willing to share" strategy. Under this circumstance, when sports enterprises actively govern and the public is willing to share, the government’s involvement can be moderately reduced and a "loose regulation" strategy can be adopted to reduce regulatory costs, improve regulatory efficiency, and create deterrence. However, if the cost of sports data processing C2 of sports enterprises is only slightly lower than the profitability of sports data of sports enterprises, sports enterprises may still violate the rules, and the loose regulation of the government provides opportunities for them.

### Simulated analysis

To verify the validity of the analysis of the stability of tripartite evolutionary game in sports data rights protection, the study assigns numerical values to the model by combining the real situation and existing research data and conducts numerical simulations using MATLAB R2017a to analyze the dynamic evolution trajectory of the equilibrium point of the tripartite evolutionary game by conducting numerical simulations for different situations respectively. Firstly, the study draws on the settings of relevant parameters in the studies of Dong, CQ, and Wei Yihua to set the initial willingness of the game subjects into three levels: high, medium, and low [13, 24]. It is uniformly notated as x, y, z ∈ (0.2, 0.5, 0.7), and the values of other parameters are formulated on this basis.

#### Scenario 1

**Fig 1:**
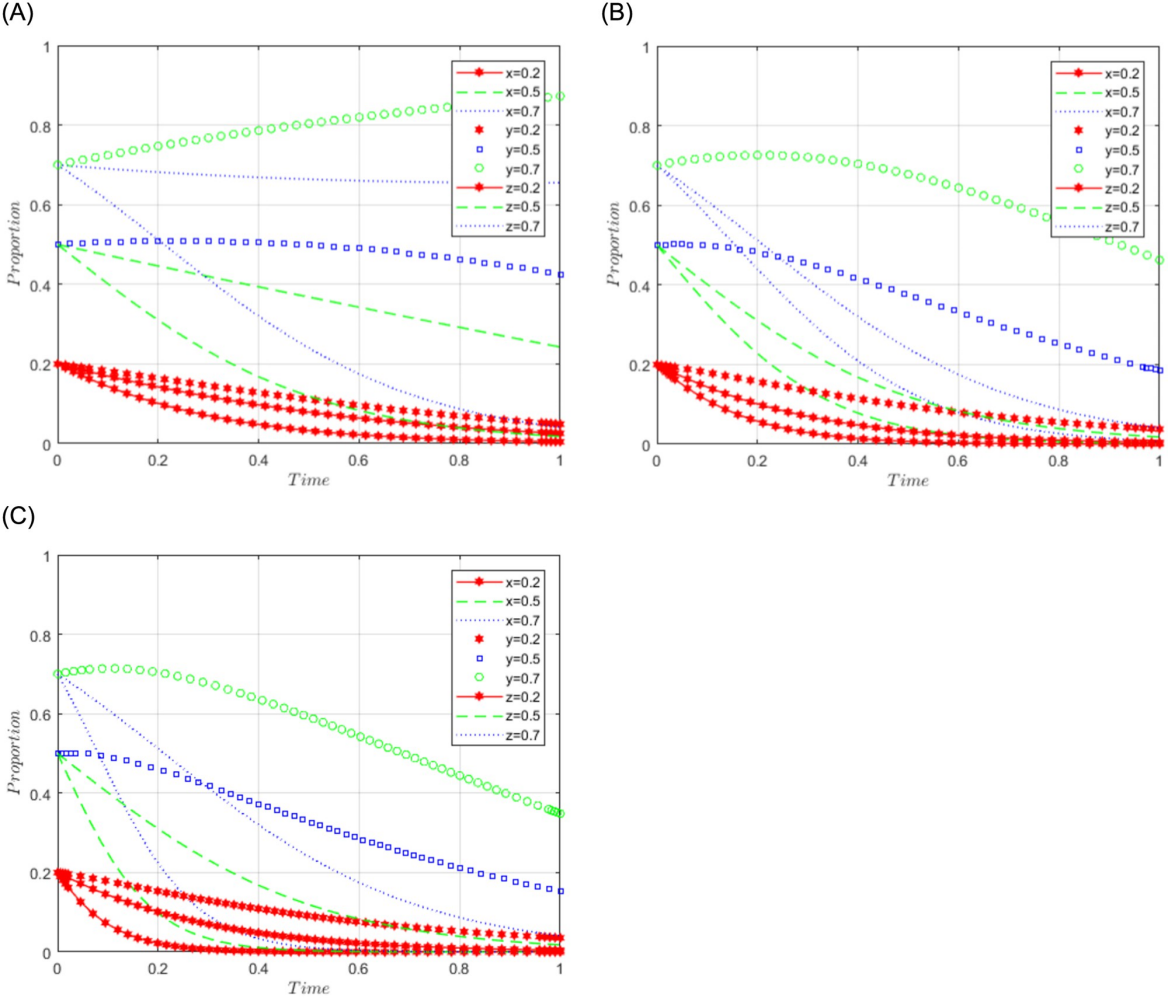
Simulation diagram of the tripartite dynamic evolution under scenario 1 (C2 = 5, 10, 15).

**Fig 2:**
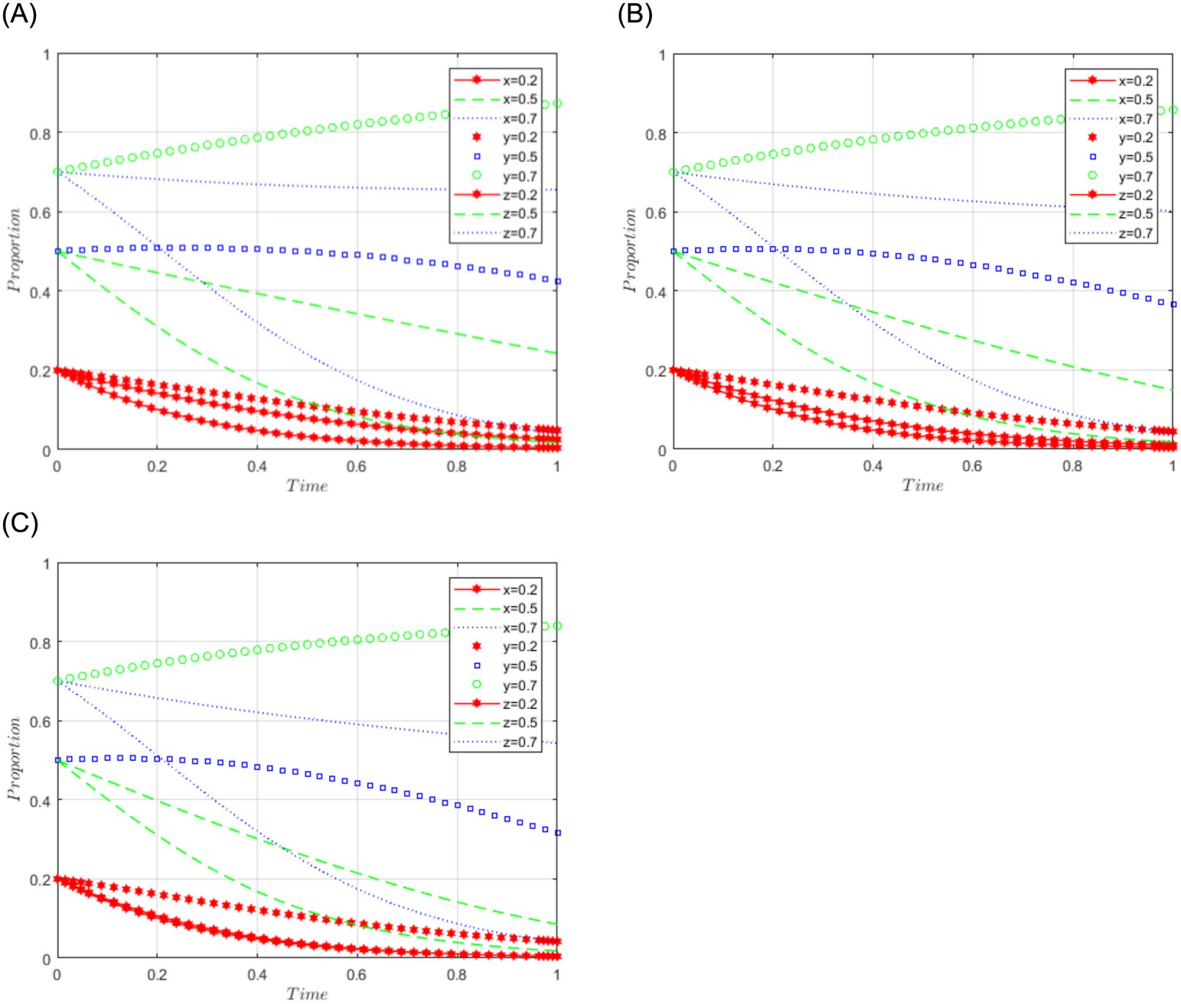
Simulation diagram of the tripartite dynamic evolution under scenario 1 (F = 10, 15, 30).

**Fig 3:**
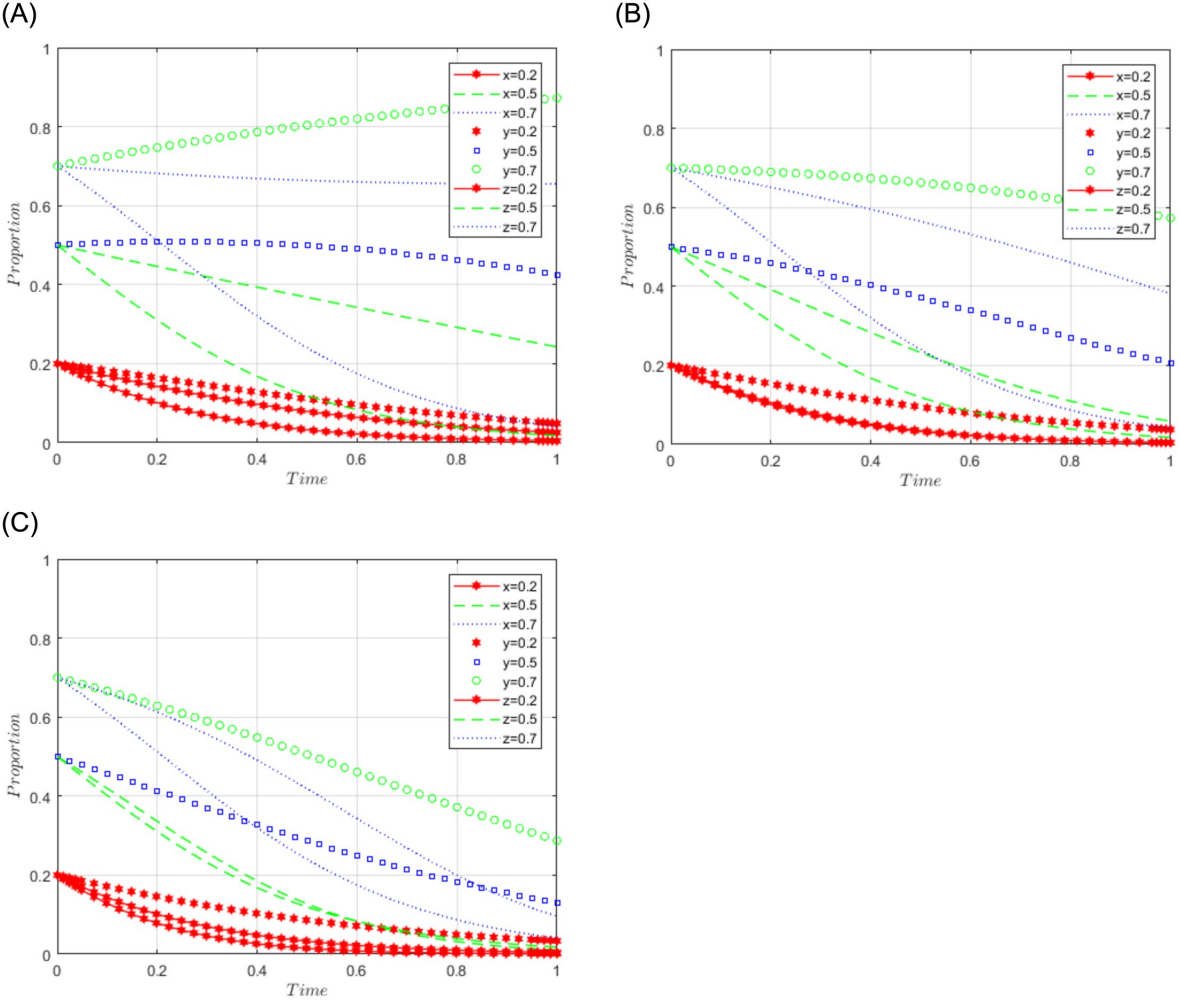
Simulation diagram of the tripartite dynamic evolution under scenario 1 (I = 10, 20, 30).

R1 = 3, R2 = 7, R3 = 5, R4 = 2, C = 5, J = 2, C1 = 2, C2 = 5, C3 = 4, L = 3, F = 5, I = 10, s = 0.5, v = 0.8, n = 0.2, m = 0.4, r1 = 0.5, l1 = 0.1, r2 = 0.4, l2 = 0.2. The set of values satisfies the condition (i) in scenario 1, and for further analysis, the effects of C2, F, and I changes on the process and results of the evolutionary game, C2 = 5, 10, 15; F = 10, 15, 30; I = 10; 20; 30 are assigned respectively, and the simulation results are shown in Figs 1–3 below. Among them, the influence of C2 changes on the process and result of evolutionary game is shown in Fig 1; the influence of F changes on the process and result of evolutionary game is shown in Fig 2; the influence of I changes on the process and result of evolutionary game is shown in Fig 3.

When x = y = z = 0.2, the game evolves along the path of scenario 1 (negative governance, refusal to share, loose regulation) and eventually converges to the game stability point D1 (0, 0, 0). When x = 0.5 or 0.7, i.e., when the initial willingness of sports enterprises is at a high level, the game evolves in the direction of scenario 1, indicating that the initial willingness of sports enterprises does not affect the evolutionary trend of the game. However, when y = z = 0.5 or 0.7, it can be found that the game does not converge to the stable point (0, 0, 0), indicating that the high initial willingness of the public and the government will produce different evolutionary directions. Further examining the influence of the changes of C2, F, and I on the evolutionary trend, it can be found from Figs 1 and 3 that the changes of C2 and I have a greater influence on the evolutionary trend of the tripartite game, which makes the game path accelerate the convergence towards the stable point. From Fig 2, it can be found that the change of F has less influence on the evolutionary trend of the tripartite game, and the tendency of the game path to converge to the stable point is not obvious. It can be seen that the higher the cost of sports data processing (C2) of sports enterprises, the more the three parties of sports enterprises, the public, and the government tend to choose the strategy of (negative governance, refusal to share, loose regulation). Facing the high cost of sports data processing, enterprises often have the problem of "illegal" use of sports data, while the government often adopts a loose regulatory model to reduce regulatory costs and improve regulatory efficiency, which makes the problems of sports data leakage and illegal use of sports data frequent, and the public’s willingness to actively share data is often greatly reduced. Even if sports enterprises pay high compensation (I) for the illegal use of the public’s sports data, they cannot influence the public’s attitude of refusing to share because the credibility of the sports enterprises has already suffered losses. In this case, an increase in fines (F) imposed by the government on sports enterprises may make sports enterprises more reluctant to participate in the use and management of sports data but has little impact on the strategic choice of the social public.

#### Scenario 2

**Fig 4:**
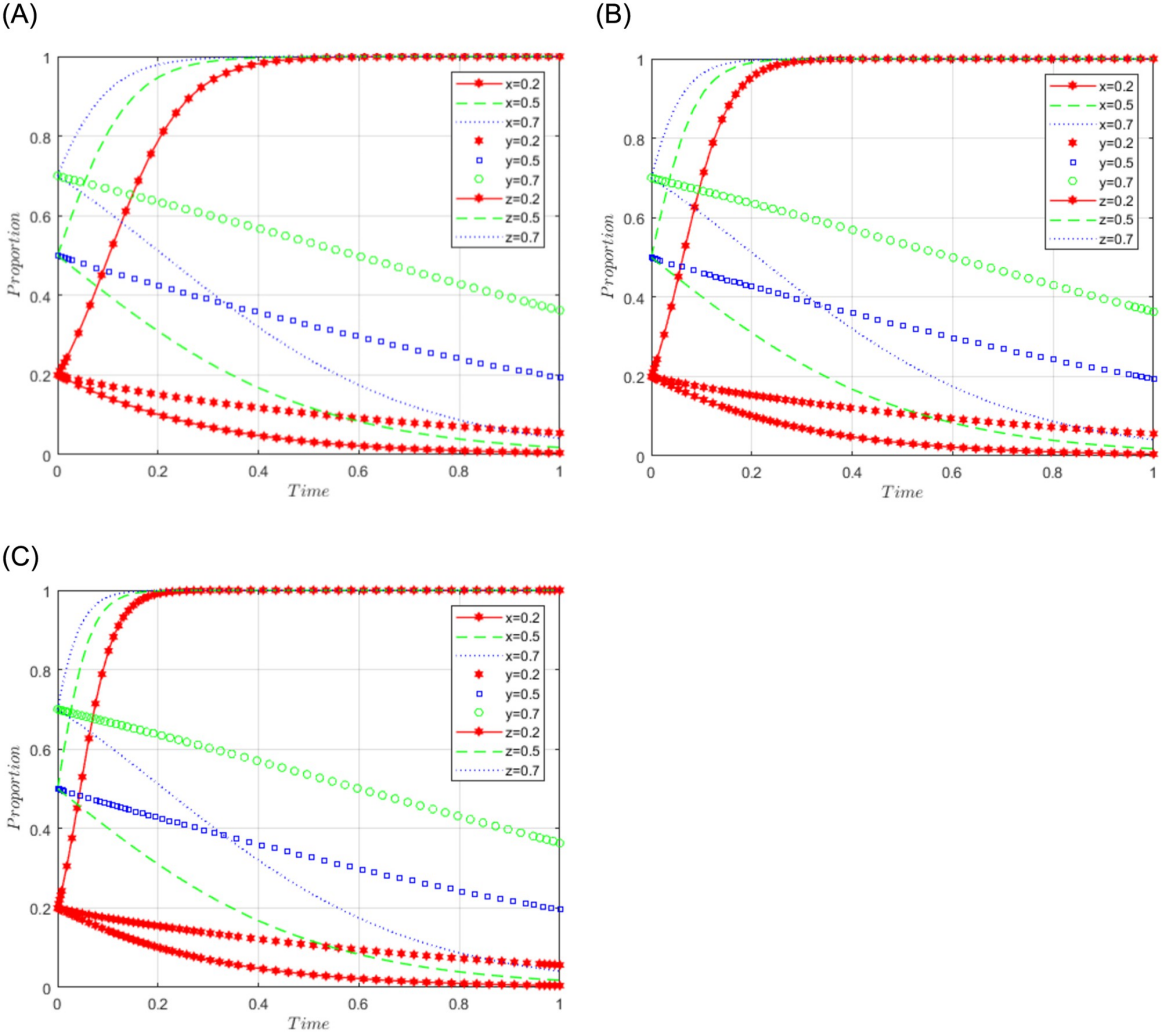
Simulation diagram of the tripartite dynamic evolution under scenario 2 (R4 = 10, 15, 20).

**Fig 5:**
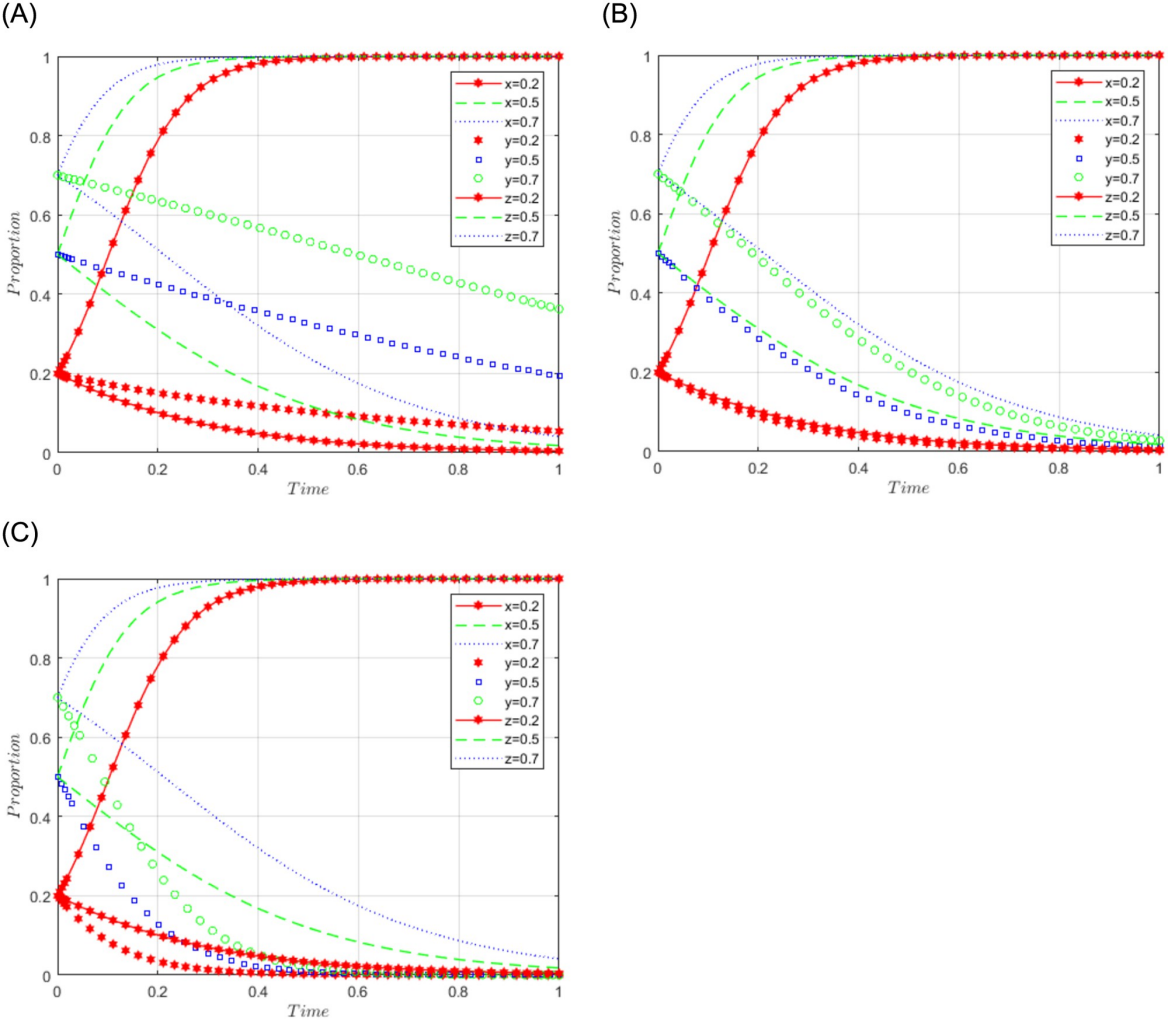
Simulation diagram of the tripartite dynamic evolution under scenario 2 (C1 = 2, 5, 10).

**Fig 6:**
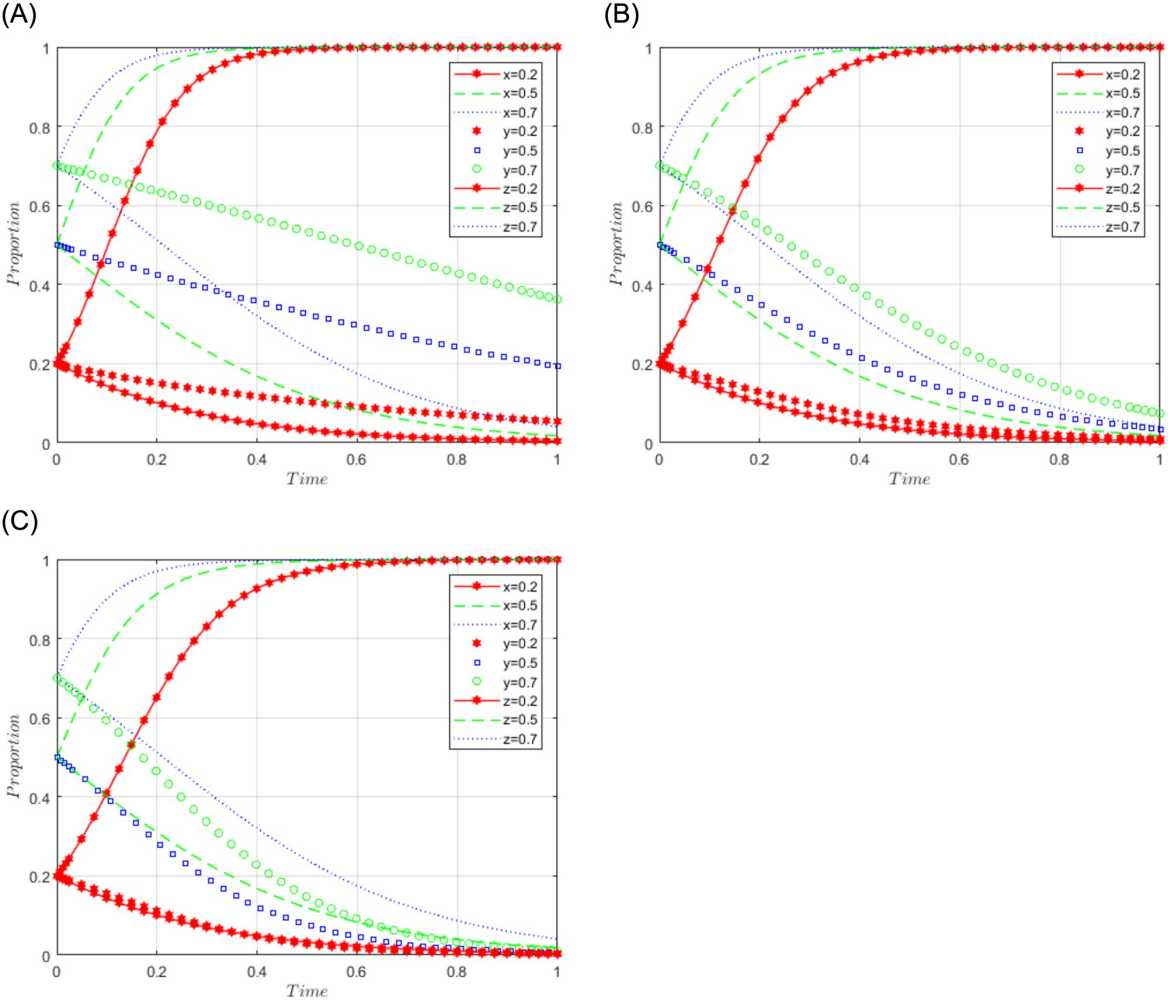
Simulation diagram of the tripartite dynamic evolution under scenario 2 (I = 10, 20, 30).

**Fig 7:**
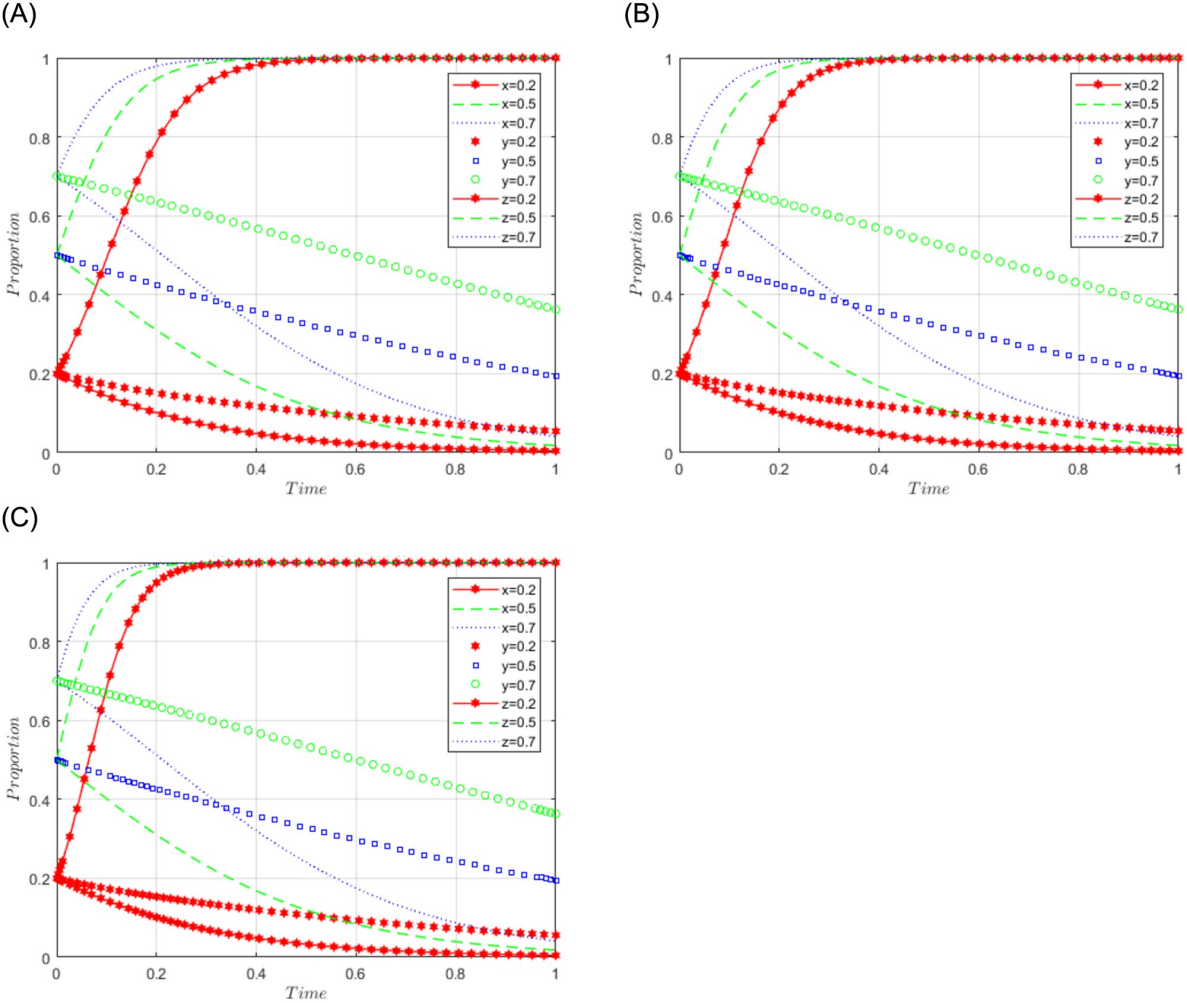
Simulation diagram of the tripartite dynamic evolution under scenario 2 (J = 2, 5, 10).

R1 = 3, R2 = 2, R3 = 5, R4 = 10, C = 5, J = 2, C1 = 2, C2 = 3, C3 = 4, L = 1, F = 5, I = 10, s = 0.5, v = 0.8, n = 0.2, m = 0.4, r1 = 0.5, l1 = 0.1, r2 = 0.4, l2 = 0.2. This set of values satisfies conditions (ii) and (iii) in scenario 2. To further analyze the effects of R4, C1, I, and J changes on the process and results of the evolutionary game, R4 = 10, 15, 20; C1 = 2, 5, 10; I = 10; 20; 30; J = 2, 5, 10, are assigned respectively, and the simulation results will be obtained as shown in Figs 4–7 below. Among them, the effect of R4 change on the process and result of the evolutionary game is shown in Fig 4; the effect of C1 change on the process and result of the evolutionary game is shown in Fig 5; the effect of I change on the process and result of the evolutionary game is shown in Fig 6; the effect of J change on the process and result of the evolutionary game is shown in Fig 7.

When the initial willingness of sports enterprises, the public, and the government is low (x = y = z = 0.2), the game will evolve along the path of scenario 2 (active governance, refusal to share, loose regulation) and eventually converge to the game stability point D2 (1, 0, 0). When x = 0.5 or 0.7, the initial willingness of sports enterprises is at a high level and their game still evolves toward active governance, indicating that the sum of the revenue level of sports enterprises and government subsidies is sufficient to motivate sports enterprises to engage in active governance. When z = 0.5 or 0.7, the government game still evolves and converges in the direction of loose regulation, suggesting that the government strategy will gradually lose regulation as sports firms actively participate in data governance. However, it can be found that the game strategy of the social public does not converge in the direction of refusing to share (when y = 0.5 or 0.7), indicating that the social public tends to be willing to share at first, while some factors may make its willingness change and evolve in the direction of refusing to share. Further examining the effects of changes in R4, C1, I, and J on the evolutionary trend, it can be found in Figs 4 and 7 that changes in R4 and J only have a more pronounced effect on the evolutionary trend of the game for sports enterprises, which accelerates the convergence in the direction of positive governance. From Figs 5 and 6, it can be found that changes in C1 and I have a greater impact on the evolutionary trend of the game for the social public and accelerate the convergence of the social public’s strategy in the direction of refusing to share. Thus, it can be seen that sports enterprises will be more actively involved in the governance of sports data with the increase in the revenue they receive (R4) and government subsidies (J). In contrast, as the cost of sharing data (C1) and the amount of compensation (I) for irregular use of data by sports enterprises increase, the strategic choice of the social public tends to refuse to share, suggesting that the main reasons influencing the social public to share data may be the higher cost of sharing and the possibility of irregular use of sports data.

#### Scenario 3

**Fig 8:**
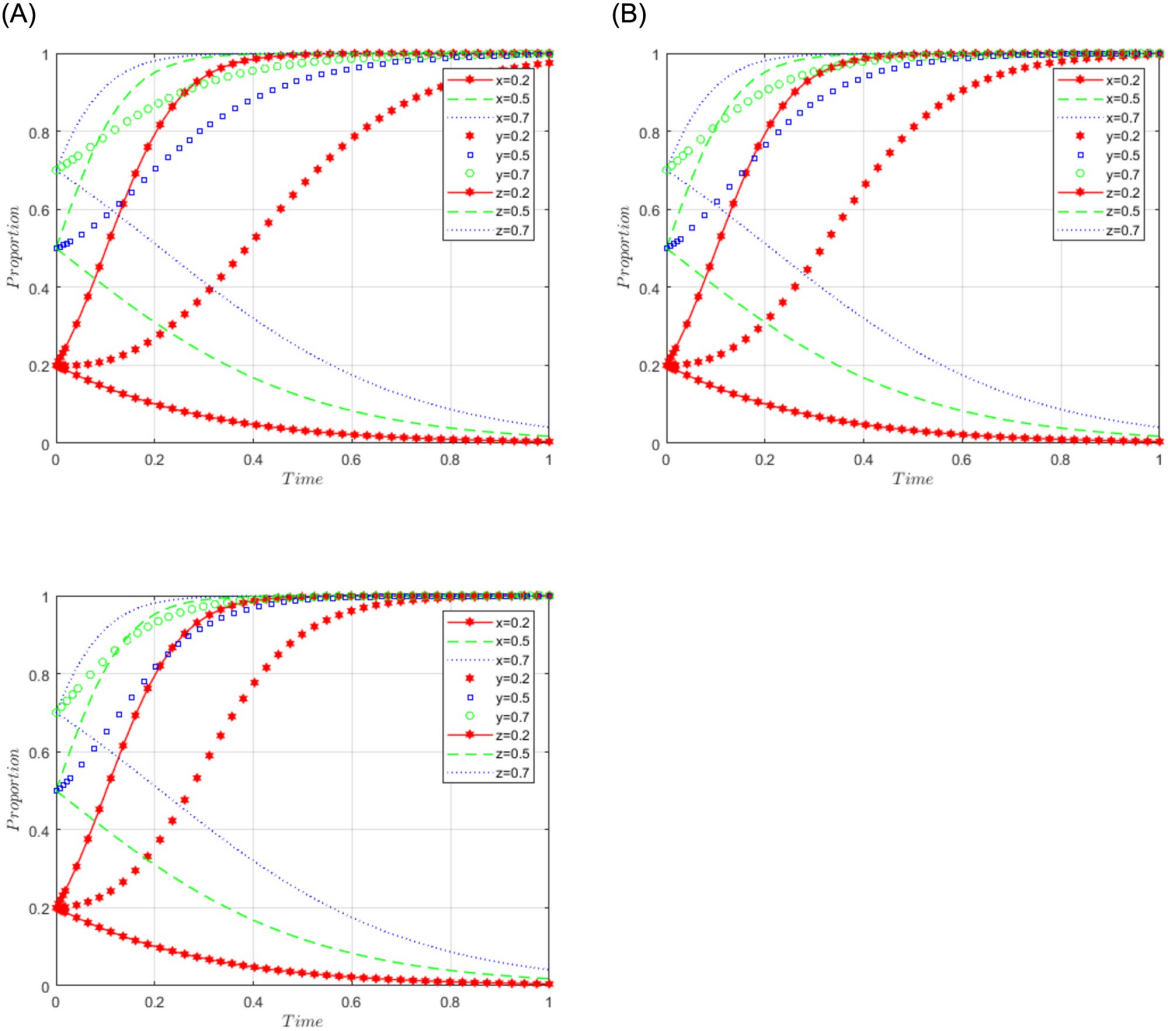
Simulation diagram of the tripartite dynamic evolution under scenario 3 (R2 = 10, 15, 20).

**Fig 9:**
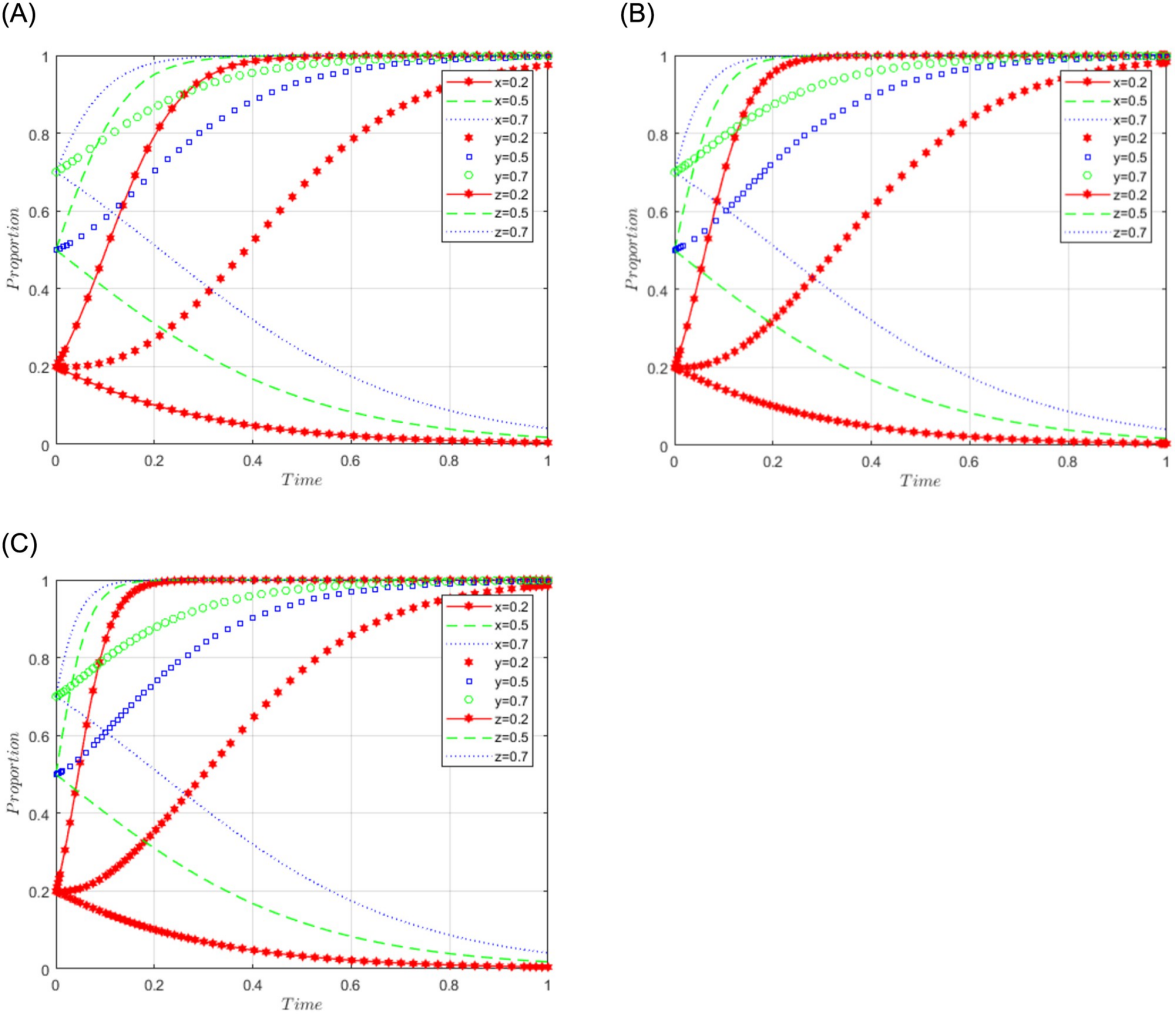
Simulation diagram of the tripartite dynamic evolution under scenario 3 (R4 = 10, 15, 20).

**Fig 10:**
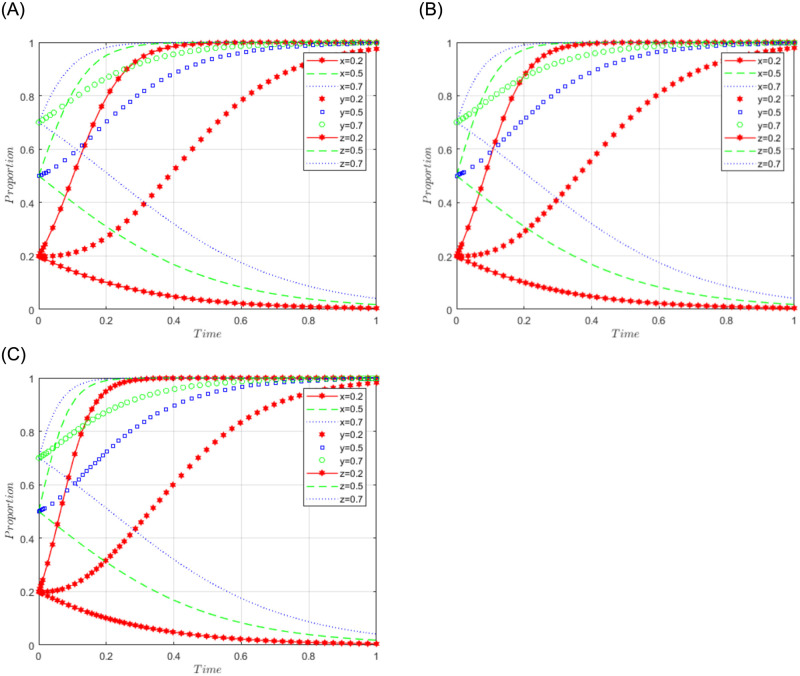
Simulation diagram of the tripartite dynamic evolution under scenario 3 (J = 2, 5, 10).

R1 = 3, R2 = 10, R3 = 5, R4 = 10, C = 5, J = 2, C1 = 2, C2 = 3, C3 = 5, L = 3, F = 5, I = 6, s = 0.5, v = 0.8, n = 0.2, m = 0.4, r1 = 0.5, l1 = 0.1, r2 = 0.4, l2 = 0.2. This set of values satisfies conditions (iv) and (v) in scenario 3. To further analyze the effects of R2, R4, C3, and J changes on the process and results of the evolutionary game, R2 = R4 = 10, 15, and 20; J = 2, 5, 10, are assigned respectively, and the simulation results will be obtained as shown in Figs 8–10 below. Among them, the influence of R2 change on the process and result of the evolutionary game is shown in Fig 8; the influence of R4 change on the process and result of the evolutionary game is shown in Fig 9; the influence of J change on the process and result of the evolutionary game is shown in Fig 10.

The dynamic evolution simulation shows that the game evolves along the path of scenario 3 (active governance, willing to share, and loose regulation) as the values of x, y, and z change, and finally converge to the game stability point D5 (1, 1, 0). This indicates that regardless of the initial willingness level of sports enterprises, their strategy tends to actively govern when the sum of revenue and government subsidies is greater than the cost of processing sports data. Similarly, regardless of the initial willingness level of the public, their strategy tends to be willing to share when the revenue is greater than the cost of sharing data. Under the benign data-sharing relationship between sports enterprises and the public, the government tends to favor lenient regulation. Further examining the influence of R2, R4, and J on the evolutionary trend, we found in Figs 8 and 9 that the convergence of the game curves of the public and the sports enterprises accelerates with the increase of R2 and R4, and both of them converge towards the direction of willingness to share and active governance. On the contrary, we found in Fig 10 that the increase of J only affects the game curves of the sports enterprises, which accelerates the convergence of the game curves of the sports enterprises. It can be seen that when the benefits (R2, R4) obtained by the public and sports enterprises are much larger than the costs to be paid, their game strategies tend to evolve and converge in the positive direction, and when the government subsidy (J) increases, it can further motivate the sports enterprises to actively participate in governance, thus realizing the virtuous development of active governance by sports enterprises, public participation in data sharing, and necessary government regulation.

## Discussion

In the practice of digital transformation, sports data presents cross-border characteristics, which makes the relationship between relevant subjects of interest intricate and complex. How to effectively identify the key influencing factors among the subjects of interest related to the protection of sports data rights, and promote the circulation and utilization of sports data are the focus of scholars’ attention. The Chinese government has been exploring relevant legislation, corporate governance, and mechanism design for the protection of data rights and interests, but there is a lack of research on the protection of sports data rights. Therefore, there is a need to design a mechanism to improve the strategic choices of different participants and to promote the circulation and utilization of sports data among various subjects. Among the existing studies, most of them are from the academic perspective, such as public management, governmental governance, regulation construction, etc., and pay less attention to the dynamic game relationship among different subjects. Therefore, we use the evolutionary game model to analyze the strategy evolution of the subjects of interest related to the protection of sports data rights, which is of theoretical value to improve the circulation and utilization of sports data, the government’s public service capacity, and the sustainable development of data. Compared with previous studies, the most important feature of our work is the use of game models and simulation tools to analyze sports data rights protection issues. However, our analytical framework focuses on horizontal analysis, and future research could be conducted simultaneously from both horizontal and vertical latitudes.

### Strengthen government regulation: Avoiding the lack of protection of sports personal data rights

Regardless of the cost of data processing and the size of the sports business, enterprises will engage in "speculative" behavior and try to avoid government regulation. In the Internet environment, large enterprises only need a small investment to obtain a large amount of data and information, at this time, the enterprise’s protection of user data is often weak [25]. Sokol and Comerford argue that monopolies relying on a dominant position in the data market are likely to control data resources and gain additional benefits to the detriment of consumers [26]. Some small and medium-sized sports enterprises (SMEs) are under even more pressure in terms of data processing, and the Tikkinen-Piri study found that compliance with the General Data Protection Regulation (GDPR) will have a significant impact on information-intensive SMEs, which may not necessarily be able to afford legal help to comply with the new regulations under the GDPR [27]. At this time, small and medium-sized sports enterprises are often more prone to violations. In this regard, government regulation becomes a key factor [28]. In terms of the regulatory approach, the government’s regulatory efforts should be flexibly adjusted to the different strategic choices of sports enterprises. For sports enterprises with more mature digital technology and richer sports resources, whose costs of sports data processing can be controlled at a lower level and who actively participate in sports data governance, the government can adopt a lenient regulatory approach. Considering the existence of market monopoly of large sports enterprises, reference can be made to the data agreement between Google DeepMind and the National Health Service (NHS) of the United Kingdom, i.e. "creative compliance", to realize the protection of data [29, 30]. However, for some traditional sports enterprises or sports enterprises with weak technology and resources, the cost of sports data processing may be at a higher level, and the government needs to strengthen regulations to avoid the "irregular" use of sports data. To prevent further negative governance attitudes among sports enterprises, the government could increase corporate subsidies to create incentives. In terms of regulatory means, the government should adopt an "all-round, all-cycle, all-chain" regulatory model, starting from business and data identification, and carry out all-round regulation for different industries (competition and performance, fitness and leisure, etc.) and different types of data (sports personal data, sports business data, sports public data) in the sports service industry; starting from the production process of sports data, the government should conduct a comprehensive regulation of sports data [31]. Starting from the production process of sports data, the whole cycle of sports data collection, sports data storage, sports data integration, sports data deep processing, sports data analysis and application, sports data archiving and processing is regulated. For the protection of sports data rights, the whole chain of supervision should be carried out in all aspects involving the use of sports data, including the provisions of the sports data confidentiality agreement beforehand, the implementation of the provisions, and the use of sports data during the process, and the circulation and sharing of sports data afterward.

### Improve the reward and punishment mechanism: Forming an effective external incentive

When the sum of revenue and government subsidies obtained by sports enterprises in the market is greater than the cost of sports data processing, sports enterprises mostly adopt a positive attitude to sports data governance. At this time, the government appropriately increases the subsidies or rewards for sports enterprises, which will further stimulate the positive behavior of sports enterprises, such as the Jiangsu Provincial Sports Bureau issued the "2023 Jiangsu Sports Industry Development Special Funds Project Announcement" to reward digital transformation and digital development projects such as sports equipment manufacturing innovation (digital sports industry) and sports integration innovation. However, there is greater uncertainty as to whether sports enterprises have a positive governance attitude if their revenues in the market are much lower than the cost of data processing and overwhelmingly dependent on government subsidies. In particular, some service-oriented, non-technology-based small and medium-sized sports enterprises tend to have a negative attitude toward sports data governance [27]. In this regard, the government needs to adopt a "targeted trickle irrigation" support model to provide targeted subsidies or incentives, and large sports enterprises should actively play a demonstration role to build digital platforms and drive collaborative innovation in the industry chain. However, the incentive mechanism alone is not enough to form an effective regulatory path for sports data rights protection and a corresponding punishment mechanism is needed to protect the rights of sports data subjects. On the one hand, when individuals initiate litigation over the improper use of sports personal data, they may face the situation that the compensation is not enough to cover the litigation costs because the value density of sports personal data is low and the actual loss is often difficult to calculate precisely, making judicial pricing often deviate from the normal price [5]. On the other hand, sports data tends to be conducted in private, with infringements being very covert, with a low probability of detection, and difficult to forensically prove [32]. In this regard, the government needs to further improve the judicial design, such as considering the principle of presumption of fault with reversal of proof, i.e., giving the burden of proof to the data processor; establishing a punitive damage system, i.e., establishing a data security assessment system by combining the degree and duration of infringement by the data processor, the sensitivity of the victim’s data, and the incremental benefit of the infringing data [33]. It was found that the promotion effect of punishment on corporate innovation significantly improved corporate performance [34]. Therefore, by improving the reward and punishment mechanism for sports data rights protection, it can form effective external incentives, and then promote the formation of an internal data governance mechanism for sports enterprises.

### Form internal data governance mechanisms: Guiding sports enterprises to improve the security level of sports data

When the risk of loss is too great after the public agrees to share, the public tends to "refuse to share" regardless of whether the sports enterprises increase the compensation or reduce the cost of data sharing. In this case, only by improving their own data security and constructing internal data governance mechanisms can sports enterprises enhance public trust and competitiveness in the sports market [35]. However, at this stage, data governance in enterprises is still mostly informal, and regulations are vague and generalized, lacking systematic support. Al-Ruithe presents recent advances in data governance in a structured, methodical, and rigorous manner, providing a guide to advancing the practice of data management [36] First, the protection of sports personal data should be the core concept of data governance for sports enterprises, which should follow the governance concept of "privacy design", make sports data rights protection the basis for the design of corporate systems and procedures, include sports data privacy protection in the highest corporate programmatic documents, and propose ethical standards. Second, sports enterprises should establish corresponding data management systems for data life cycle management, data quality management, and other areas, including sports data compliance organization system, sports data compliance risk process, sports data compliance review process, sports data violation reporting, and handling process and other management systems, to supervise and execute sports data management of sports enterprises. Since sports data of sports enterprises include sports commercial data produced by themselves and also include collected sports personal data and open sports public data, they need to be managed hierarchically and categorically according to different sports data types, such as management of user data (sports personal data), functional department data (sports commercial data), data managed by third parties (sports public data), data involving personal rights, and management of emergency events (cyber attacks, data theft, etc.). Third, improper data management may have a significant negative impact on the organization [37]. Therefore, there is a need to build a preventive system for sports data protection and take preventive measures against possible data security problems. This includes regular or irregular risk assessment reviews, such as differentiated assessment and safeguards according to the application scenarios of sports data; comprehensive investigation and analysis of departments or businesses with data security risks, which may be dangerous due to the uncertainty of the regulation of the secondary use of data [38]. Therefore, there is a need to investigate the departments or operations involved in the work of secondary dissemination and utilization of personal data in sports, resulting in a risk report Finally, sports enterprises should build an internal sports data monitoring system, including supervision and auditing of sports data, in which supervision of sports data processing, handling, storage and sharing should be included in the scope of responsibility of managers to check whether there is any improper use or violation of sports data; auditing requires the audit department to independently review sports data processing and use, thus ensuring that sports enterprises form an effective internal data governance mechanism and are well implemented.

## Conclusions and limitations

### Conclusions

From the perspective of stakeholders, this study constructs a tripartite evolutionary game model of the government, sports enterprises, and the public in sports data protection, and uses numerical simulation to further study the idealized "cooperation" mode of the stakeholders in sports data protection. The study shows that the ideal evolutionary equilibrium of the model is the active governance of sports enterprises, the participation of the public in data sharing, and the necessary regulation by the government. However, the cost of sports data governance, the cost of sports data sharing, the degree of compliance in the use of sports data, the government’s incentive mechanism, and the government’s regulatory mechanism are the key factors influencing the behavioral decisions of the three parties in the game. If the government departments through the establishment of appropriate reward and punishment mechanism measures, will motivate sports enterprises to actively improve the whole process of sports data compliance governance and regulatory system, thereby enhancing the credibility level of sports enterprises in the public, prompting the public to actively participate in the sharing and circulation of sports data, and ultimately will converge to the evolutionary stability strategy of active governance, willingness to share, and lax regulation.

### Main contributions

The main contribution of this study is reflected in both theoretical and practical dimensions.

From the perspective of theoretical development, unlike most previous studies that focused on violations of sports event network broadcasting, sports event data ownership, and sports personal data privacy and security issues, this study considers discussing the multi-interested subject relationship involved in sports data as the entry point of the study. Second, this study introduces the evolutionary game model into the study of sports data rights protection and provides a new research framework and research path. The advantage of the evolutionary game over traditional game is that it combines game theory and dynamic evolution process analysis. This makes our simulation results consistent with realistic situations because the idealized canonical model does not exist in reality. Finally, we use the simulation analysis to verify that there are steady-state strategy points in the tripartite evolutionary game model, and this result provides a basis for further cracking the dilemma of sports data rights protection through mechanism design.

From the perspective of practical significance and social impact, the practical significance of our work is mainly reflected in three aspects: cooperation among relevant stakeholders, institutional strength, and mechanism exploration. First, mutual trust and cooperation among relevant stakeholders can play a positive role in the protection of sports data rights and lay the foundation for the circulation and utilization of sports data. Second, the findings of this study provide direction and support for government departments and sports enterprises to manage sports data. Third, exploring and promoting characteristic mechanisms are also practical initiatives supported by the findings of this study, including strengthening government regulation, improving reward and punishment mechanisms, and forming internal data governance mechanisms for enterprises.

### Limitations and future research

The characteristics of sports data rights protection are not the same at different stages. Thus, the game should not only be dynamic but can also be a multistage model, where the set of strategies at each stage may not be consistent. In addition, according to the classification of the sports industry, the types of sports data are more diverse and the game models of relevant interest subjects are also different. Therefore, we suggest that future research should be conducted in three aspects. First, scholars can construct a multi-stage game model based on life cycle theory, which will help to further improve the game process. Second, the risk preference characteristics of relevant interest subjects should be considered, and prospect theory can be introduced to explain the impact of subject risk preference characteristics on behavioral decisions. Third, the relationship of relevant subjects’ interest in different types of sports data should be considered, and the game model can be constructed for different types of sports data and further analysis can be carried out.

## Supporting information

S1 Data Minimal data. (DOCX)
