# Supplementary material for: A tripartite evolutionary game analysis of sports data rights protection from the perspective of stakeholder
Source: PLoS One. 2023 Nov 16;18(11):e0292914. doi: 10.1371/journal.pone.0292914 (PMC10653474; doi:10.1371/journal.pone.0292914)
Supplement: S1 Data — (DOCX) [file pone.0292914.s001.docx]

**Minimal Data**

**x, y, z ∈ (0.2, 0.5, 0.7)**

1. R1=3, R2=7, R3=5, R4=2, C=5, J=2, C1=2, C2=5, C3=4, L=3, F=5, I=10, s=0.5, v=0.8, n=0.2, m=0.4, r1=0.5, l1=0.1, r2=0.4, l2=0.2
2. R1=3, R2=2, R3=5, R4=10, C=5, J=2, C1=2, C2=3, C3=4, L=1, F=5, I=10, s=0.5, v=0.8, n=0.2, m=0.4, r1=0.5, l1=0.1, r2=0.4, l2=0.2
3. R1=3, R2=10, R3=5, R4=10, C=5, J=2, C1=2, C2=3, C3=5, L=3, F=5, I=6, s=0.5, v=0.8, n=0.2, m=0.4, r1=0.5, l1=0.1, r2=0.4, l2=0.2
